# Synthesis, Characterization and Sorption Capacity Examination for a Novel Hydrogel Composite Based on Gellan Gum and Graphene Oxide (GG/GO)

**DOI:** 10.3390/polym12051182

**Published:** 2020-05-21

**Authors:** Cristina Modrogan, Andreea Mădălina Pandele, Constantin Bobirică, Dan Dobrotǎ, Annette Madelene Dăncilă, Gabriel Gârleanu, Oanamari Daniela Orbuleţ, Claudia Borda, Delia Gârleanu, Cristina Orbeci

**Affiliations:** 1 Faculty of Applied Chemistry and Materials Science, Politehnica University of Bucharest, Polizu 1-7, 060042 Bucharest, Romania; c_modrogan@yahoo.com (C.M.); madalina.pandele@upb.ro (A.M.P.); annette.dancila@upb.ro (A.M.D.); oanamari.orbulet@upb.ro (O.D.O.); cristina.orbeci@upb.ro (C.O.); 2 Faculty of Engineering, Lucian Blaga University of Sibiu, 550024 Sibiu, Romania; 3 Faculty of Industrial Engineering and Robotics, Politehnica University of Bucharest, 060042 Bucharest, Romania; gabriel.garleanu@upb.ro (G.G.); claudia.borda@upb.ro (C.B.); delia.garleanu@upb.ro (D.G.)

**Keywords:** hydrogel composite, graphene oxide, gellan gum, zinc sorption

## Abstract

A novel hydrogel composite based on gellan gum and graphene oxide (GG/GO) was synthesized, characterized and tested for sorption capacity in this work. The microstructural, thermogravimetric and spectroscopic analysis confirmed the formation of the GG/GO composite. Comparative batch sorption experiments revealed a sorption capacity of the GG/GO composite for Zn (II) ions of approximately 2.3 higher than that of pure GG. The GG/GO composite exhibits a maximum sorption capacity of 272.57 mg/g at a pH of Zn (II) initial solution of 6. Generally, the sorption capacity of the sorbents is approximately 1.5 higher in slightly acidic conditions (pH 6) comparative with that for strong acidic conditions (pH 3). The sorption isotherms revealed that the sorption followed a monolayer/homogenous behavior. The sorption kinetic data were well fitted by the pseudo-second-order kinetic model, and were consistent with those derived from sorption isotherms. The intraparticle diffusion was considered to be the rate-determining step. Two main sorption mechanisms for Zn (II) were identified namely, ion exchange at low pH values, and both ion exchange and chemisorption in weekly acidic conditions.

## 1. Introduction

Along with the rapid progress in industry (i.e., metal plating facilities, mining operations, fertilizer industries, tanneries, batteries, paper, pesticides industries, etc.) the developed countries are daily facing with an increase in the discharge of heavy metals in wastewater, which poses a serious risk to public health [1]. Toxic heavy metals such as zinc, copper, nickel, mercury, cadmium, lead, arsenic, cobalt and chromium [2,3] are of particular concern in the treatment of wastewaters.

Zinc is an essential element for human health especially for the physiological functions of living tissue and for regulation of many biochemical processes. It acts as a microelement in the metabolic processes but exceeding doses may pose toxic effects. An increase in the zinc concentration in the body can cause serious health problems including stomach cramps, skin irritations, vomiting, nausea and anemia. Its toxicity depends significantly on both its speciation and target organism [4]. The World Health Organization (WHO) has set maximum allowable limits for heavy metal ions in drinking water [5,6]. The maximum allowable limit for Zn (II) is 3 mg/L [7]. Currently a wide range of techniques for removing heavy metals, including zinc, from industrial wastewater is known. Of these, the most commonly applied are chemical precipitation, ion-exchange, adsorption and membrane separation [8]. However, in many cases, besides the removal efficiency, the selectivity and the operating cost are the criteria that define the choice of the method of removing heavy metals from industrial wastewater. In this respect, biosorption appears to be one of the most attractive methods for selective removal of heavy metal ions from wastewater [9]. A wide range of biosorbents were tested but it seems that those formed into beads (i.e., polysaccharides such as gellan gum, xanthan gum, carob gum, etc.) are much more efficient than those that consist of raw materials (i.e., wheat straw, fruit, nut shells, algae, etc.) [10]. Recently, some multifunctional hybrid biosorbents were synthetized and successfully tested for contaminants removal from aqueous waste solutions [11,12]. These hybrid biosorbents are in fact reinforced hydrogels based on reinforcing fillers such as clay, carbon nanotubes, graphene oxide, etc. [13]. The reinforcing fillers offer to the hydrogels, beside an improved strength, an enhanced adsorptive capacity.

Currently, graphene and its derivatives have received special attention being intensively used in many applications in medicine, energy, electronics, detectors, environmental, etc. The entire success they enjoy derives from the unique properties (mechanical, thermal, electrical, photoelectrical, biological, catalytic, etc.) that these materials possess [14]. From the point of view of environmental applications, graphene and its derivatives have been used either alone or in combination with other materials to remove a wide range of contaminants, both organic and inorganic chemical species, from various bodies of water and wastewater [15,16]. In this respect, a series of hybrid biosorbents based on graphene oxide (GO) was used with good results to remove contaminants from aqueous solutions such as GO/cellulose hydrogel for heavy metals removal [17,18], chitin/GO hybrid composites for dyes removal [19], iron oxide/persimmon tannin/GO for rare earths removal such as erbium [20], etc.

Gellan gum is an anionic polysaccharide derived from the *Sphingomonas elodea* bacterium. Like other polysaccharides, gellan gum forms gels in the presence of monovalent or divalent metal cations, which are soft and therefore need to be reinforced with other materials in order to get suitable mechanical properties [21]. Contrary to other polysaccharides, gellan gum maintains its gel state over a large range of pH of aqueous systems [22]. This behavior gives it excellent acid stability, which makes it a very good candidate for the preparation of sorbents with applications in removing heavy metal ions from acidic aqueous solutions. In addition to acid stability, gellan gum exhibits very good thermal stability, behaves very well in solutions with high ionic strength, and reacts well with metal ions [23]. Despite these special properties, there has been little interest until now in using gellan gum in environmental applications such as removing contaminants from water and wastewater bodies. Examining the sorption capacity of gellan gum or gellan gum-based composites for contaminants, as well as identifying the sorption mechanisms involved, could help to open the way for using these materials in green water and wastewater treatment technologies.

Additionally, a possible solution for the decontamination of multi-contaminated wastewater is the use of guar gum/graphene oxide (AGG/GO) hydrogel using borax as a crosslinker, which has a high surface area with a crosslinked structure for increased removal of dyes and heavy metals. Therefore, the hydrogel thus prepared can have applications in the field of adsorption, due to its good biodegradability and durability [24].

Gellan gum is part of the large family of exopolysccharides (EPS). Gellan gum hydrates in hot water and the low acyl form also hydrates in cold water with sequestrants. On cooling, native high-acyl gellan gum gives gels that are soft and elastic. Low-acyl gellan gum gels at very low concentrations using both monovalent and divalent cations to give firm, brittle textures with excellent thermal stability. Combinations of gellan gum can be used to control syneresis and form a range of textures from soft and elastic to firm and brittle. At levels too low to form a demoldable gel, gellan gum can form fluid gels that can suspend wastewater particles. It has already been tested for mass production of EPS (i.e., one company can produce over 500 tonnes of gellan gum per year) [25].

With the aim to explore new adsorbents for water purification, guar gum based hydrogels were synthesized by cross-linking with borax at a different percentage. Thus, it has been established that hydrogels synthesized based on guar gum have high efficiency in the process of removing the blue dye Aniline. At the same time, the best hydrogel surpassed aluminum at an extremely low concentration and also showed a color removal efficiency of up to 94%. [26,27].

Examining the sorption capacity of gellan gum or gellan gum-based composites for contaminants, as well as identifying the sorption mechanisms involved, could help to open the way for using these materials in green water and wastewater treatment technologies.

Therefore, the objective of this work was to synthesize and characterize a novel hydrogel composite based on gellan gum and graphene oxide (GG/GO) followed by testing it through sorption experiments for the removal of zinc ions from aqueous solutions in different experimental conditions. It is worth mentioning that it is desired, on the one hand, that the new composite hydrogel be obtained mainly from gellan gum, which is obtained from natural products and therefore friendly to the environment, and on the other hand, to have a high efficiency for sorption of heavy metal ions, which could be reached by adding a minimum amount of graphene oxide, which also ensures a minimum manufacturing cost. Examination of the sorption capacity of the new composite hydrogel was performed on synthetic solutions of zinc ions, which are part of the heavy metal ions category that could be successfully removed by such a sorbent. The obtained results were used to establish and describe the sorption mechanisms underlying the retention of zinc ions, which can be also extended for other divalent heavy metal ions.

## 2. Materials and Methods

All reagents were of analytical grade and were used without any further purification. Zinc chloride (ZnCl_2_) and hydrochloric acid (HCl) were purchased from Sigma-Aldrich (subsidiary of Merck KGaA, Darmstadt, Germany). High-purity graphene oxide was kindly supplied by the National Institute for Research and Development in Microtechnologies (IMT, Bucharest, Romania). It was obtained from natural graphite according to the Hummers protocol [28]. The provider has checked the formation of graphene oxide by Raman spectroscopy [29]. Phytagel^TM^ (high acyl Gellan Gum) was purchased from Sigma-Aldrich (subsidiary of Merck KGaA, Darmstadt, Germany). Stock solutions of Zn (II) were prepared by dissolving zinc chloride (ZnCl_2_) in deionized water at room temperature. The working solutions were prepared by diluting the stock solution with deionized water and hydrochloric acid (HCl) used to adjust the pH to the preset values.

### 2.1. Gellan-Gum/Graphene Oxide (GG/GO) Synthesis

**Figure 1:**
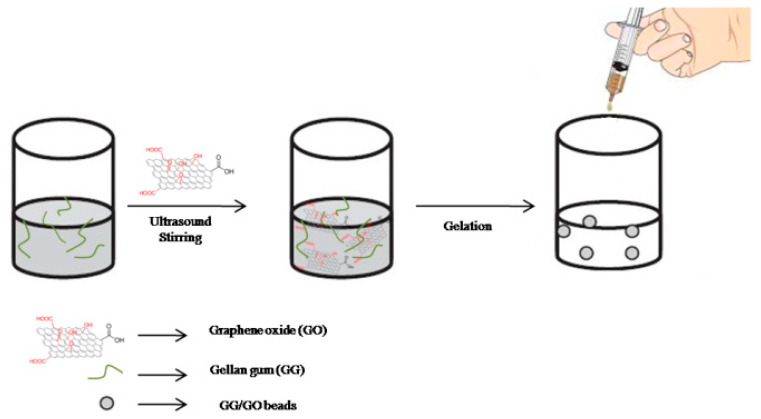
Synthesis protocol of gellan gum/graphene oxide (GG/GO) hydrogel composite beads.

The synthesis of the new composite hydrogel was accomplished by a simple gelation method described in detail in the following. Thus, 1 g of Phytagel^TM^ was dissolved in 50 mL deionized water at 80 °C under magnetic stirring to form 2 wt % gellan gum solution (GG). After cooling at 50 °C, 0.005, 0.01 and 0.02 g of graphene oxide (GO) were added in order to obtain a GO dosage of 0.5, 1 and 2 wt %, and then the mixtures were ultrasonicated for 1 h at room temperature for homogenization. Afterwards, the mixtures were added dropwise into 1 wt % calcium chloride (CaCl_2_) solution under constant and gentle stirring using a syringe of 0.85 µm inner diameter to form the beads. The obtained gelled beads were stirred for another 30 min at room temperature into CaCl_2_ solution, and then the beads were filtered, washed several times with deionized water to remove the CaCl_2_ excess and dried at room temperature for 48 h before being used. The preparation procedure is schematically shown in Figure 1.

### 2.2. Removal Efficiency of Zn (II) by GG and GG/GO Composites

It is worth mentioning that, in order to optimize the number of measurements, only the GG/GO composite for which the best results were obtained from preliminary tests in terms of Zn (II) removal efficiency was characterized and intensively tested for its sorption capacity for Zn (II). In this regard, the sorbents and Zn (II) solution with an initial concentration of 100 mg/L were mixed for 24 h at room temperature (22 ± 1 °C) under continuous agitation at a liquid-to-solid mass ratio of 50/1. Then the samples were filtered and the residual concentration of Zn (II) in the solution was measured by atomic absorption spectrometry using a spectrometer contrAA 300, Analytik Jena. The Zn (II) removal efficiency was determined according to the Equation (1).

(1) $$R=\frac{C_{i}-C_{f}}{C_{i}}\times 100$$

where *R* is the removal efficiency, and *C_i_* and *C_f_* are the initial and final concentration of Zn (II) (mg/L).

### 2.3. Sorbents Characterization

The sorbents used in the experiments were characterized before and after their use in sorption experiments. In this respect, the formation of the GG/GO composite was highlighted by Fourier-transform infrared spectroscopy-attenuated total internal reflectance (FTIR-ATR, Bruker Optik GmbH, Ettlingen, Germany) and thermogravimetric analysis (TGA, TA Instruments, New Castle, USA). The sorption of Zn (II) on both GG and GG/GO was highlighted by X-ray photoelectron spectroscopy (XPS, Thermo Fisher Scientific, Waltham, MA, USA) and scanning electron microscopy-energy dispersive X-ray analysis (SEM-EDAX, Philips, Hillsboro, OR, USA). FTIR spectra were recorded on a Bruker VERTEX 70 spectrometer using 32 scans with a resolution of 4 cm^−1^ in the wave number region of 4000–600 cm^−1^. TGA curves were recorded on a Q500 TA Instrument, in nitrogen atmosphere, in the temperature range of 20–800 °C, and at a heating rate of 10 °C/min. Both GG and GG/GO composite surface structures, before and after Zn (II) sorption were studied by XPS using a K-Alpha instrument from Thermo Scientific with a monochromatic Al Kα source (Al Kα = 1486.6 eV), at a background pressure of 2 × 10^−9^ mbar. The binding energy was corrected for surface charging by taking the C 1s peak as a reference at 284.6 eV. The pass energy was set to 200 eV for survey scans and to 20 eV for the region scans. Morphological analysis was performed on a FEI XL 30 ESEM TMP microscope equipped with an EDAX Sapphire device.

### 2.4. Sorption Isotherms and Kinetics

The sorbents and Zn (II) solution, with concentrations ranging from 5 to 100 mg/L, were mixed for 24 h at room temperature (22 ± 1 °C) under continuous agitation at a liquid-to-solid mass ratio of 50/1. Afterwards, to separate the sorbent from the aqueous solutions, the samples were filtered and the residual concentration of Zn (II) as well as the concentration of Ca (II) in the solution was measured by atomic absorption spectrometry using a spectrometer contrAA 300, Analytik Jena. The amount of Zn (II) taken up by sorbents was determined according to the Equation (2).

(2) $$q_{eq}=\frac{\left(C_{0}-C_{eq}\right)V}{m}$$

were *q_eq_* is the amount of Zn (II) taken up by sorbent at equilibrium (mg/g), *C_0_* and *C_eq_* are the initial and equilibrium concentration of Zn (II) (mg/L), V is the volume of the solution (L) and m is the mass of sorbent (g). The isotherms were drawn by plotting the amount of zinc taken up by sorbent *q_eq_* (mg/g) as a function of equilibrium concentration of zinc *C_eq_* (mg/L). To describe the types of interactions between sorbate (Zn (II)) and the sorbents as well as to determine the maximum sorption capacity of the sorbent, Langmuir and Freundlich adsorption isotherm models were used.

The experimental data for sorption kinetics were obtained by contacting the sorbents with Zn (II) solution (concentration of 100 mg/L) at room temperature (22 ± 1 °C) under continuous agitation at a liquid-to-solid mass ratio of 50/1 for 2.5, 5, 10, 20, 30 and 60 min. At the end of each contacting period the samples were filtered and the residual concentration of Zn (II) in the solution was measured by atomic absorption spectrometry using a spectrometer contrAA 300, Analytik Jena. The amount of zinc ions taken up by sorbents was determined according to the Equation (3).

(3) $$q_{t}=\frac{\left(C_{0}-C_{t}\right)\times V}{m}$$

where *q_t_* is the amount of Zn (II) taken up by sorbent at time t (mg/g), *C_0_* and *C_t_* are the initial and residual concentration of Zn (II) at time t (mg/L), V is the volume of the solution (L) and m is the mass of sorbent (g). Pseudo-first-order, pseudo-second-order and intraparticle diffusion kinetic models were used to evaluate the experimental data for sorption kinetics.

All experiments were performed in triplicate. Zn (II) ions were arbitrary chosen so that to test the sorption capacity of the new composite for divalent heavy metal ions. The experiments were performed for two pH values of Zn (II) solution, namely 3 and 6. The pH values were chosen so that to represent two extreme conditions in which the sorption experiments were to be carried out. It is well known that the carboxylate groups of the sorbents are non-ionized at a pH value less than 3.4, and become ionized at pH values higher than 4.4. The occurred negative charges will cause the swelling of the sorbents matrix due to the expansion of the polymer chain with a maximum at a pH value of 7.4 [30]. It is expected that both the ionization and swelling degree to have a high influence on the sorption capacity of the sorbents. Although it seems that the maximum swelling degree of the matrix is reached near neutral pH conditions, however in order to avoid a possible precipitation of zinc ions during experiments, a pH value corresponding to slightly acidic conditions was set in this work (pH 6). Therefore the experiments regarding the sorption of zinc ions will be carried out under two extreme working conditions: the most unfavorable (pH 3) and the most favorable (pH 6) conditions.

### 2.5. Swelling Measurements

To determine the swelling characteristic, the sorbents were first dried until constant mass was reached, weighed and then immersed for 24 h in phosphate buffer solution corresponding to the two pH values used. Then, the samples were removed from the solution, filtered and weighed. The degree of swelling was calculated using the formula presented below.

(4) $$\%\;S=\frac{\left(wet\;mass-dry\;mass\right)}{dry\;mass}\times 100$$

## 3. Results and Discussions

### 3.1. Removal Efficiency of Zn (II) by GG and GG/GO Composites

**Figure 2:**
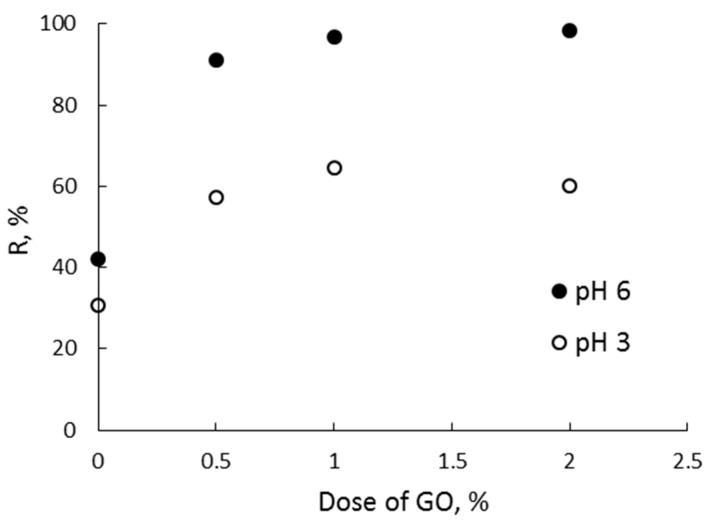
Removal efficiency of Zn (II) by the synthetized sorbents.

Figure 2 shows the Zn (II) removal efficiency as a function of the dose of GO added to the composite hydrogel synthesis mixture. It can be clearly seen that the removal efficiency of Zn (II) sharply increased with the addition of up to 1% GO, but became practically insignificant, or even decreased in acidic conditions at pH 3, at a higher GO dose. In this respect, in slightly acidic conditions at pH 6, the removal efficiency increased from 42% for pure GG to approximately 97% for GG/GO 1 wt % composite. Next, adding 1% more GO led to an increase with only 1%, the removal efficiency reaching approximately 97%. In acidic conditions at pH 3, the removal efficiency increased from approximately 31% for pure GG to approximately 64% for GG/GO 1 wt % composite, and then decreased to approximately 60%. These results highlighted two distinct aspects related to the Zn (II) removal efficiency, namely, the major difference between the results obtained at the two extreme pH values regardless of the dose of GO, which is related to the sorption mechanisms, and the optimal dose of GO, which seems to be 1% regardless of the pH value. Therefore, to optimize the number of experiments and measurements, only pure GG and GG/GO composite 1 wt % will be characterized and extensively tested at the two pH extreme values of 3 and 6 to establish the sorption capacity and sorption mechanisms of Zn (II). For ease of writing, hereinafter the composite GG/GO 1 wt % will be written as GG/GO.

### 3.2. FTIR Spectroscopy

**Figure 3:**
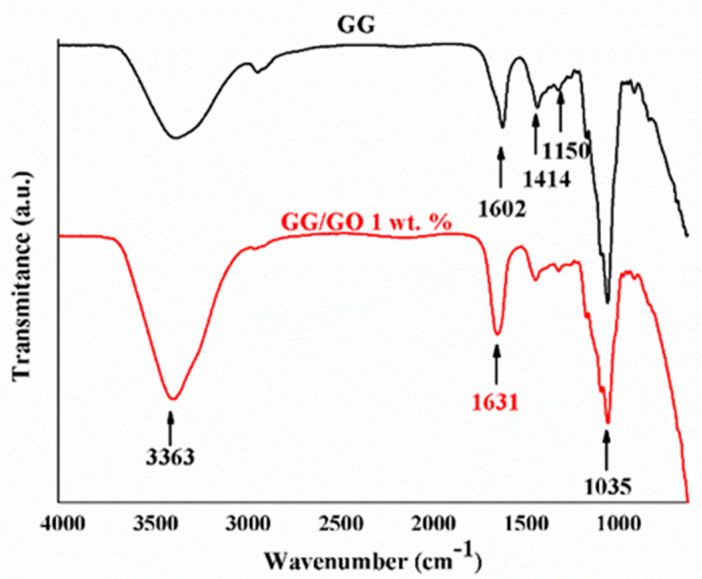
FTIR spectra of GG and GG/GO hydrogel composite beads.

The FTIR spectrum of GG (Figure 3) exhibited a broad band at 3363 cm^−1^ assigned to the hydrogen groups from glucopyranose ring. The band at 2925 cm^−1^ was attributed to the stretching vibration of –CH_2_ groups. The presence of the COO^−^ group was observed at 1602 and 1414 cm^−1^ respectively. The bands at 1150 and 1035 cm^−1^ corresponded to the ethereal and hydroxylic C-O stretching [31]. Compared with GG spectrum, the GG/GO beads spectrum (Figure 2) displayed the same peaks as the pure polymer but with a significant shift of the band assigned to COO^−^ group from 1602 to 1631 cm^−1^ indicating the formation of some interfacial hydrogen bonds between GO and the polymer. Moreover, sp^2^ hybridization took place for each carbon atom in GO, which further contributes to the remaining electrons in the p orbital in order to obtain the delocalized π bond. This led to a free movement of π electrons although the hydroxyl group of the GG chain could increase the π-electron density of the conjugated system and presents an electron-donation effect inducing an increase of the adhesion between the two compounds [32]. The FTIR spectra evidenced the interaction between GO nanosheets and the polymer chain of GG and a close adhesion between the two compounds.

### 3.3. TGA Analysis

**Figure 4:**
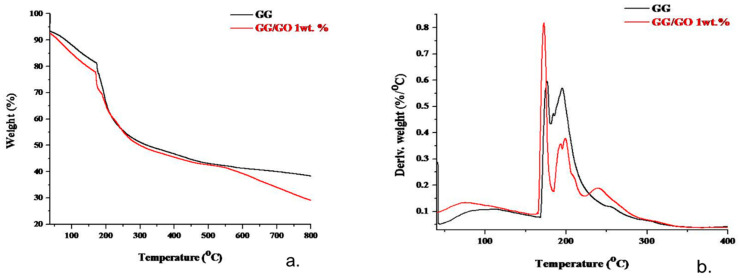
TGA (**a**) and DTG (**b**) curves of GG and GG/GO hydrogel composite beads.

In order to study the thermal stability of GG and composite beads the thermogravimetric analysis (TGA) was conducted. Figure 4 exhibited the TGA (Figure 4a) and DTG (Figure 4b) curves of pure GG and the GG/GO composite beads. Both samples indicate a weight loss around 80 °C due to the evaporation of the trapped and physical adsorbed water molecules in the sample [33,34]. The next degradation steps between 170 and 300 °C were assigned to the decomposition of the polymer backbone and to the oxygen-containing functional groups including hydroxyl, epoxy, carboxyl and carbonyl group from the GO surface. The sharp exothermic effect with a maximum around 170 °C in the DTG curve of GG/GO is specific to graphene oxide from the composite whose oxidation when it is in the pure form occurs suddenly with high intensity near the temperature of 155 °C [35]. However, shifting the maximum of this thermal effect towards higher temperatures in the case of the GG/GO composite reflects a chemical modification of GO, which is most probably due to the interaction with GG. Moreover, unlike the pure GG, in the case of GG/GO composite, a new degradation step can be observed at about 240 °C (Figure 4b), which is attributed to the degradation of the functional groups attached to the GO sheet edges. It has been proven that those functional groups are more thermally stable compared with the one attached to the basal aromatic plane of the GO sheet [32]. According to the thermogravimetric curves profile, it appears that the GG/GO composite was slightly less thermal stable compared with pure GG. This could be due to the improving the thermal conductivity of GG after the addition of GO, which enhanced the heat transfer rate in the composite. In this respect, the decomposition rate of GG in the composite is accelerated and results in a higher weight loss rate of GG/GO comparative with pure GG [36,37].

### 3.4. XPS Analysis

**Figure 5:**
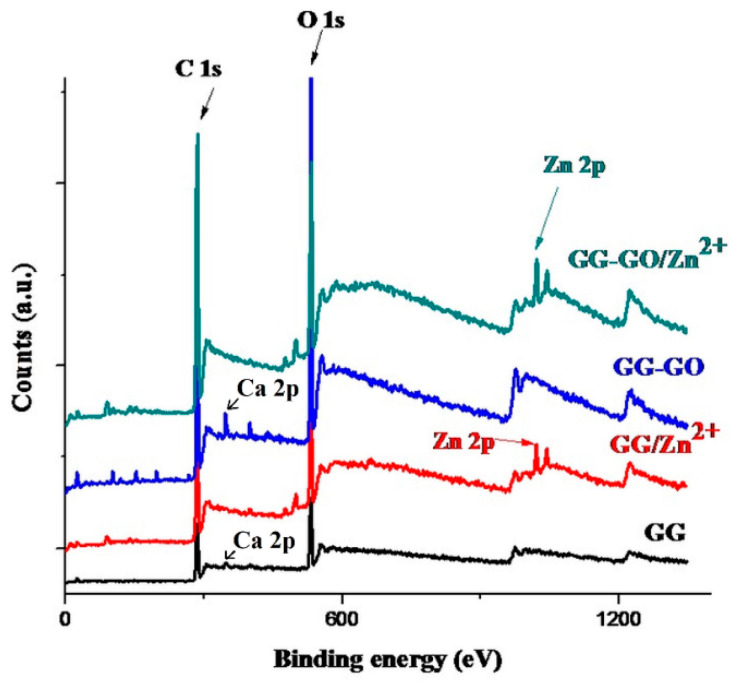
XPS spectra for GG; GG/GO before and after sorption of Zn (II).

Figure 5 shows XPS survey spectra of GG and GG/GO before and after Zn (II) sorption. As can be seen, besides the C 1s and O 1s peaks, specific to the polymer framework, a Zn 2p peak appeared in the both GG and GG/GO spectra after sorption. The appearance of zinc in the spectra after sorption is strong proof that the sorption process occurred. Moreover, after a close examination of the data in Table 1, GG-GO composite beads exhibited a slight increase in Zn (II) concentration compared to pure GG beads. In addition, the Ca 2p peak that appears in both GG and GG/GO spectra before sorption is missing in their spectra after sorption. These results bring valuable information on the sorption mechanism of Zn (II), which is discussed in the next sections of this work.

### 3.5. SEM-EDAX Analysis

**Figure 6:**
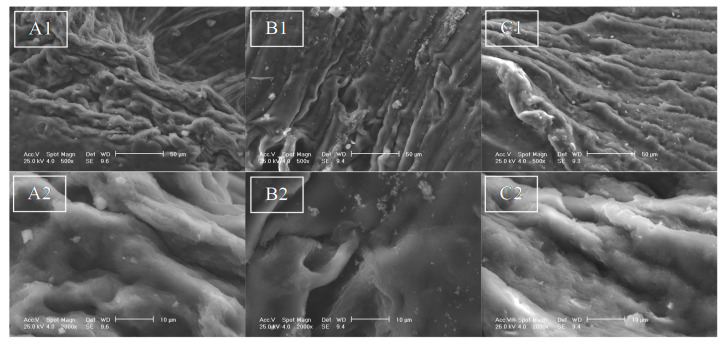
SEM images of GG (**A**), GG with sorbed Zn (II) (**B**) and GG/GO with sorbed Zn (II) (**C**). Index 1 is for 500× magnification and index 2 for 2000× magnification.

**Figure 7:**
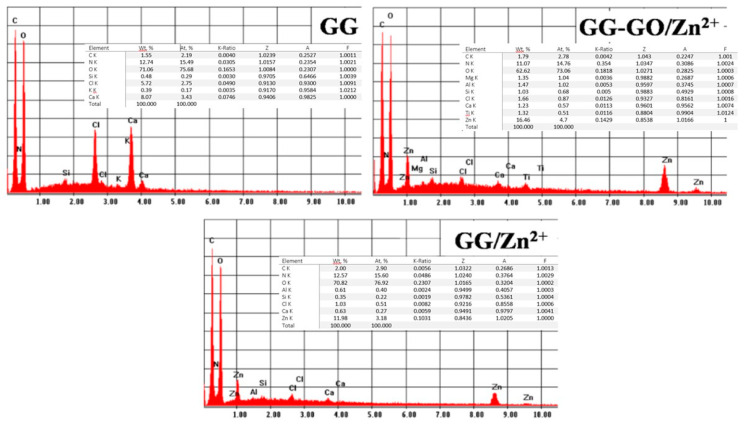
Energy dispersive X-ray analysis (EDAX) spectra of GG and GG/GO beads before and after Zn (II) sorption.

The changes in the morphology of the composite hydrogel compared to the starting hydrogel, as well as the highlighting of the retention of Zn (II) ions, were studied by SEM-EDX analysis (Figure 6 and Figure 7). A careful analysis of the micrograph of the composite hydrogel (Figure 6C1) revealed the presence of a much higher number of more small cavities in its inner structure compared to the structure of GG. It is known that this type of structure favors the absorption of water and, therefore, the swelling properties of the material. This may be just one of the reasons why composite hydrogel (GG/GO) had a much higher swelling degree than polymer (GG), as will be seen below. Additionally, the structure of the GG/GO composite (Figure 6C2) looked a bit more compact and rough than that of the GG polymer, which could be the result of adding of GO whose slices get intercalated into the GG polymer structure [38]. These findings highlight that the addition of GO influence the network structure and change the surface morphology of GG.

From EDAX spectra (Figure 7) it could be observed that after the sorption of Zn (II), only C and O peaks in the both GG and GG/GO spectra remained but instead, some new peaks assigned to Zn (II) emerged. The presence of the other elements in the spectra was assigned to the gelation process and to the water that was at the site where the beads were was stored until used. These results were in accordance with those obtained by XPS analysis.

### 3.6. Sorption Isotherms

**Figure 8:**
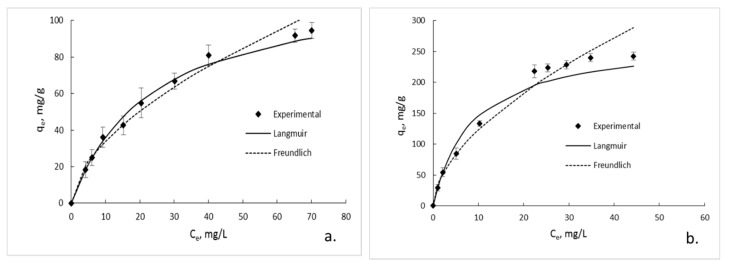

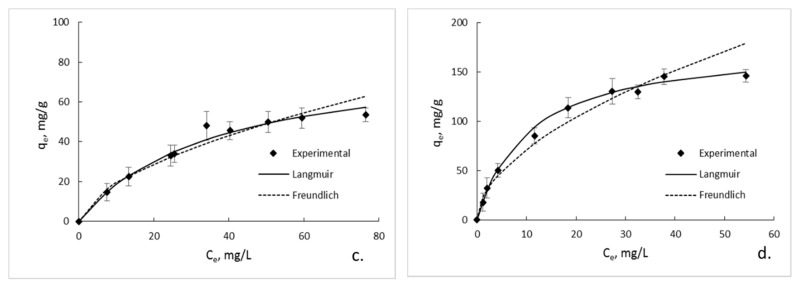
Sorption isotherms of Zn (II) from aqueous solutions under different pH conditions: (**a**). GG at pH 6; (**b**). GG/GO at pH 6; (**c**). GG at pH 3 and (**d**). GG/GO at pH 3. Error bars represent the calculated standard deviation for the experimental data.

Sorption isotherms of Zn (II) sorbed onto GG and GG/GO at pH 3 and 6 are shown in Figure 8. As can be seen, the sorption capacity of both GG and GG/GO increased as the initial concentration of Zn (II) increased. Moreover, the sorption capacity is always higher in weekly acidic conditions. In this respect, at pH 6 and Zn (II) initial concentration of 100 mg/L, the experimental maximum sorption capacity was 120.48 mg/g for GG and 272.27 mg/g for GG/GO, while at pH 3 and the same Zn (II) initial concentration, the experimental maximum adsorption capacity was 84.03 mg/g for GG and 178.57 mg/g for GG/GO (Table 2). The differences may be due to the availability of functional oxygen groups in the structure of the sorbent materials, which mainly depended on the environmental conditions (e.g., pH, ionic strength, etc.), as well as to the type of interaction between the zinc ions and the surface of these materials. To better illustrate the performance of GG/GO composite for sorption of Zn (II), a comparison with other sorbent materials presented in the literature [38,39,40,41,42,43] was made (Table 3). As can be seen, the sorption capacity of GG/GO composite for Zn (II) obtained in this work has comparative value with those presented in the literature.

To further understand the interaction between the sorbate, (i.e., Zn (II)), and the sorbent surface (GG or GG/GO), the obtained experimental data were fitted with typical Langmuir (Equation (5)) and Freundlich (Equation (6)) isotherm models. The regression parameters are presented in Table 2. The Langmuir model assumed monolayer coverage on a surface with energetically homogenous identic binding sites with no interaction between adsorbed species. The Freundlich model assumes a heterogeneous surface with an exponential distribution of the bonding sites and their energies [40].

(5) $$\frac{C_{e}}{q_{e}}=\frac{1}{K_{L}}+\frac{C_{e}}{q_{m}}$$

(6) $$q_{e}=K_{F}\times C_{e}^{1/n}$$

*q_e_* in the above equations is the sorption capacity at equilibrium (mg Zn/g of sorbent), *K_L_* is the Langmuir sorption constant (L/mg) and K_F_ is the Freundlich sorption constant (mg^1−n^·L^n^/g) related to the energy of sorption, *C_e_* is the equilibrium concentration of Zn (II) in the liquid phase (mg/L), q_m_ is the maximum sorption capacity (mg Zn/g of sorbent) and 1/n is the dimensionless heterogeneity factor.

According to the quadratic value of correlation coefficients (R^2^) the sorption of Zn (II) was better evaluated by the Langmuir isotherm model than the Freundlich one for both sorbents. Therefore it can be concluded that the sorption of Zn (II) on the GG and GG/GO sorbents followed monolayer/homogenous behavior. Although the sorption process did not follow the Freundlich isotherm model well, the n values were found to be greater than 1 (Table 2), which indicates favorable conditions for sorption of Zn (II) on both GG and GG/GO sorbents.

### 3.7. Sorption Kinetics

**Figure 9:**
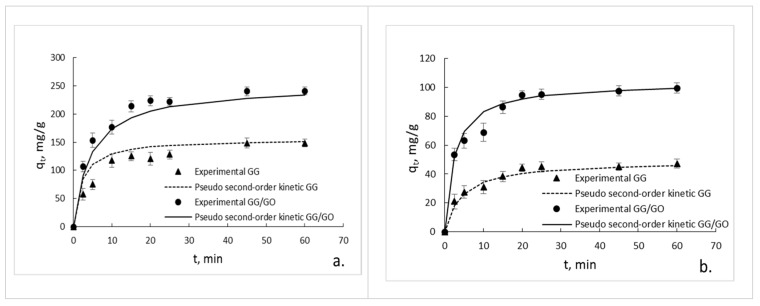
Kinetic curves for Zn (II) sorption on GG and GG/GO sorbents from aqueous solutions under different pH conditions: (**a**). GG and GG/GO at pH 6; (**b**). GG and GG/GO at pH 3.

The experimental kinetic curves for Zn (II) sorption on GG and GG/GO sorbents are presented in Figure 9. As can be seen, in all experiments the sorption reached equilibrium in approximately 20 min, and the tendency is that the maximum sorption capacity is higher under slightly acidic conditions (Table 4). The results are consistent with those derived from sorption isotherms (Figure 8). Pseudo-first-order, pseudo-second-order and intraparticle diffusion model were used to evaluate the kinetic sorption data. Linear forms of these kinetic models are presented in Equations (7)–(9) [44,45,46]. The kinetic parameters and the correlation coefficients are presented in Table 4.

(7) $$ln\left(q_{e}-q_{t}\right)=ln\;q_{e}-k_{1}t$$

(8) $$\frac{t}{q_{t}}=\frac{1}{k_{2}q_{e}{}^{2}}+\frac{t}{q_{e}}$$

(9) $$q_{t}=K_{id}t^{1/2}+C$$

q_e_ and q_t_ in the above equations are the sorption capacity at equilibrium and at time t, respectively, (g Zn (II)/g of sorbent), k_1_ is the pseudo-first-order rate constant (min^−1^), k_2_ is the pseudo-second-order rate constant (g mg^−1^ min^−1^), K_id_ is the diffusion rate coefficient (mg g^−1^ min^−1/2^) and C is the intercept and it is related to the thickness of the boundary layer.

According to the correlation coefficients (R^2^) the kinetic sorption of Zn (II) was better evaluated by the pseudo-second-order kinetic model for all experiments. The kinetic rate (k_2_) is always higher for GG/GO and in the slightly acidic conditions (Table 4). These results are indicative for a higher affinity between Zn (II) and the available and well distributed active sorption sites of GG/GO beads, which leads to a faster sorption. In addition, the sorption capacity of GG/GO beads follows the same trend suggesting that the high surface area of GO and the existence of oxygenous functional groups on its surface have an important role in increasing the sorption capacity.

**Figure 10:**
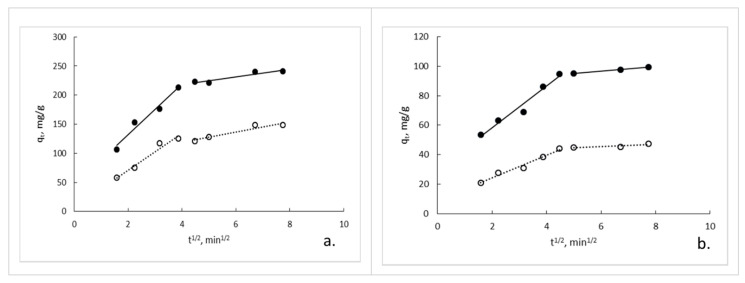
Intraparticle diffusion model for Zn (II) sorption from aqueous solutions under different pH conditions: (**a**). GG and GG/GO at pH 6; (**b**). GG and GG/GO at pH 3.

The rate controlling steps of the sorption process were analyzed by plotting the sorption capacity at time t (q_t_) versus the square root of the sorption time (t^0.5^). The plots are shown in Figure 10 and the parameters of the intraparticle diffusion model, including the correlation coefficients (R^2^), are presented in Table 4. As can be seen, the plots were not linear over the entire timeframe, and presented two main sections of linearity that did not pass through the origin. These results suggest that there are two major rate controlling steps of the sorption process. First, external diffusion of the Zn (II) takes place (boundary surface diffusion), and second, intraparticle diffusion of the Zn (II) takes place (the diffusion of Zn (II) in the intraparticles spaces and pores of the sorbent) [47]. The linear sections were much more flattened on plots (Figure 10), which are associated with the intraparticle diffusion step, suggesting that this diffusion step could be considered as a rate-determining step.

### 3.8. Sorption Mechanism

**Scheme 1:**
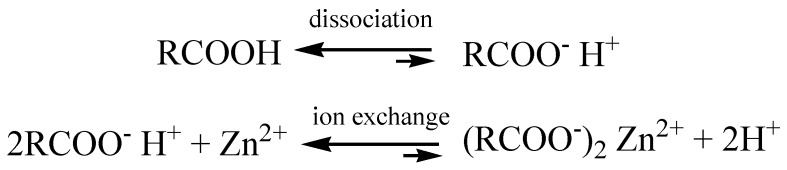
Behavior of the carboxyl groups under acidic conditions.

The pH of the aqueous solution plays an important role in the sorption process of the heavy metal ions, including zinc, because it influences both the form of the heavy metal in the aqueous solution and the form in which the available functional groups of the sorbent are present [48]. At pH lower than 7.0, Zn (II) is the dominant dissolved-zinc species [49]. On the other hand, at low pH values (pH < 4.0), the concentration of H^+^ ions is high and the protons compete with Zn (II) ions for oxygenous functional groups (e.g., hydroxyl and carboxyl) resulting in their protonation. In addition, the presence of carboxyl groups in the structure of both GG and GG/GO makes them behave like weak acid ion exchangers. In their acid form the carboxylic acid functional groups (–COOH) are, on the one hand, poorly dissociated and, on the other hand, the high affinity of the carboxyl groups for free hydrogen ions that exist in the solution greatly shifts the equilibrium in favor of their association. This behavior makes almost impossible an ion exchange with other cations in the solution [50]. The processes described above are represented in Scheme 1 (the short equilibrium arrow signifies the unfavorable path of the process).

**Figure 11:**
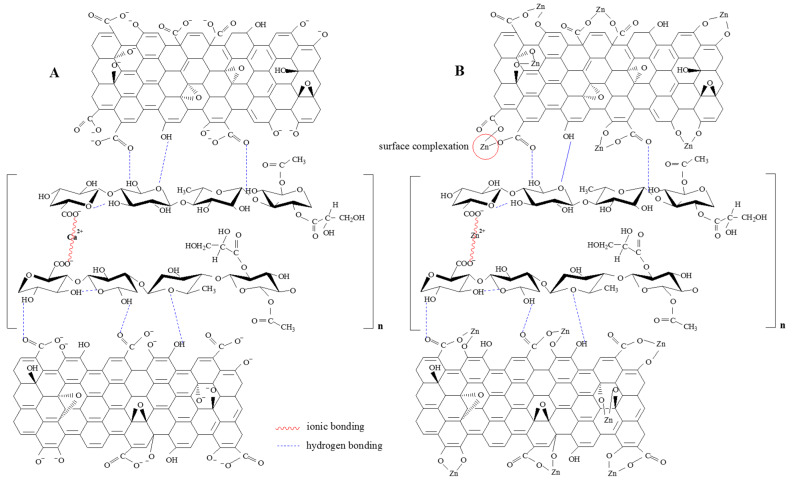
Schematic representation of Zn (II) sorption on GG/GO at pH 6. (**A**): before sorption; (**B**): after sorption.

This may be one of the reasons why the sorption capacity of both sorbents was lower at low pH. On the contrary, in weekly acidic conditions (pH > 5.0) the oxygenous functional groups are mostly deprotonated and the surface of the sorbents is negatively charged, Zn (II) ions being able to interact electrostatically with these. Once arrived in close proximity of the oxygenous functional groups through the electrostatically driving force, Zn (II) ions can react, on the one hand, with oxygenous functional groups located both at the basal plane and at the edges of the GO nanosheets, and on the other hand, with available oxygenous functional groups from the structure of GG to form mainly inner-sphere complexes. The speculated sorption sites of Zn (II) on the GG/GO composite are schematically represented in Figure 11. The negatively charged surface of the sorbents can be highlighted by electrokinetic potential (zeta potential) measurements [51,52]. In this respect, pure GG has a zeta potential ranging from −10 to −31 mV for pH between 3 and 7 [53]. Additionally, pure GO has a zeta potential ranging from −30 to −40 mV for pH between 3 and 6 [54].

The polymeric chains of GG are cross-linked through an ionic bonding between Ca (II) (from CaCl_2_ used as a cross-linking agent) and the carboxylate anions of the D-glucuronosyl residues located on the two different tetrasaccharide repeating units. The intermolecular Ca (II) bridge between the polymeric chains of GG is confirmed by XPS (Figure 5) and EDAX (Figure 7) analysis. The cross-linked GG interacts with GO probably through intermolecular hydrogen bonding mainly between hydroxylic and carboxylic functional groups of the cross-linked GG and GO, respectively. Although the presence of intermolecular hydrogen bonds cannot be definitely associated with the broad band between 3000 and 3700 cm^−1^ (Figure 3), which is usually assigned to the free and associated hydroxyl (O–H) stretching vibrations from COOH, C–OH and H_2_O, the considerable increase in the intensity of this band in the GG/GO spectra could be a sign of their formation (Figure 3).

**Scheme 2:**
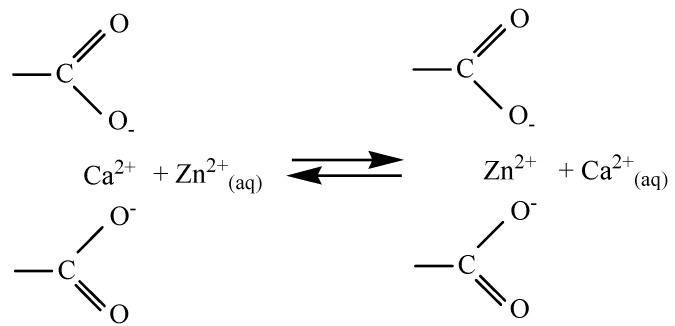
Ion exchange between the intermolecular Ca (II) bridges and Zn (II).

During the sorption process, calcium ions from the intermolecular Ca (II) bridges are exchanged with zinc ions leading to the formation of intermolecular Zn (II) bridges. This ion-exchange reaction can also take place at low pH values (pH < 4.0). The process is depicted in the Scheme 2.

**Scheme 3:**
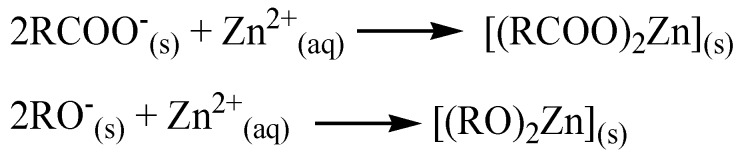
The chemisorption of Zn (II).

This process is confirmed by XPS (Figure 5) and EDAX (Figure 7) analysis. As can be seen from the XPS spectra of both GG and GG/GO after sorption, the specific peaks for calcium, initially (before sorption) present in the two spectra, are missing, while new peaks associated with zinc appear in both of them. New zinc-specific peaks occurring in both XPS and EDAX spectra confirm the retention of Zn (II) through sorption processes that are primarily ion exchange at low pH values and both ion exchange and chemisorption (surface complexation) in weekly acidic conditions. The chemisorption of Zn (II) is depicted in the Scheme 3.

**Figure 12:**
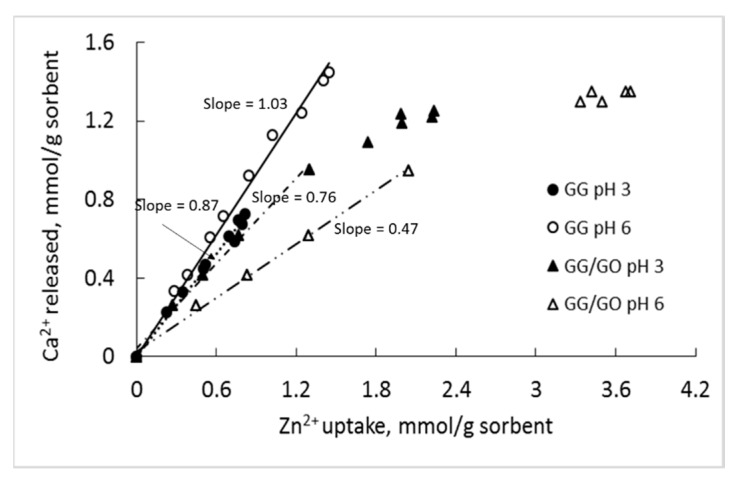
Relationship between zinc ions uptake by sorbents and calcium ions released from the sorbents.

To support the above explanation, the relationship between the amount of zinc ions uptake by sorbents and the calcium ions released from the sorbents (the intermolecular Ca (II) bridge between the polymeric chains of GG) in the solution is shown in Figure 12. For GG there is a linear dependence on the whole range of zinc concentration in the initial solution regardless of its pH value. The slope of the regression line is very close to unity, which indicates a stoichiometric relationship between the two variables, which is described by the ion-exchange reaction presented above. In the case of GG/GO, the relationship between the two variables is linear only for a low concentration of zinc ions in the initial solution regardless of its pH value, and it has the tendency to flatten out as the zinc ions concentration increases. However, the slope of the regression line on the linear section was far from unity compared to GG. This behavior might suggest another zinc ions uptake mechanism such as surface complexation (as presented above), which is mainly associated with the graphene oxide (GO).

In this sense, it must be taken into account that GG swelling has a pH-dependent behavior with maximum swelling below neutral pH. [55].This behavior was also demonstrated experimentally in this work by determining the degree of swelling according to the pH values used. In this sense, the swelling degree of GG and GG/GO at pH 3 was 168% and 232%, respectively. The degree of swelling of GG and GG/GO at pH 6 was 1013% and 2178%, respectively. Therefore, the diminished sorption capacity of both GG and GG/GO for Zn (II) at low pH values (pH < 4.0) could be attributed to low swelling of GG and subsequently to the reduction in their surface area. On the other hand, the presence of GO nanosheets in the structure of the GG/GO increased both its overall surface area and its potential for interaction of Zn (II) with the available oxygenous functional groups from the structure of GO, which led to a substantial increase of its sorption capacity. This may be the reason why GG/GO always had a higher sorption capacity than GG.

### 3.9. Desorption and Reusability Behaviors of GG/GO

The applicability potential of GG/GO composite from both economic and technical standpoint derives from the possibilities of its regeneration, which could led to the possibility to reuse it in successive sorption/desorption cycles. The reuse of used sorbents and the selective recovery of metal ions are two significant features that must be considered in order to establish the efficiency of newly developed materials [56,57,58].

Both desorption and reusability experiments were performed on the GG/GO composite under the conditions in which the highest sorption capacity was obtained, namely under slightly acidic conditions at pH 6. Three sorption/desorption cycles were performed. The results obtained in terms of desorption efficiency were promising, in the sense that this parameter had a value of 57% after the first desorption cycle, and after the following two desorption cycles, the desorption efficiency value did not decrease much (52% after the second cycle, and 50% after the third cycle). This demonstrates the high efficiency and the practical application potential of this type of sorbent.

## 4. Conclusions

A novel hydrogel composite based on gellan gum and graphene oxide (GG/GO) was synthesized in this work through a simple gelation method. The obtained material was characterized through microstructural, thermogravimetric and spectroscopic analysis, and was tested for sorption capacity of Zn (II) ions. The results revealed the formation of the GG/GO composite. The batch experiments revealed a maximum sorption capacity of the GG/GO composite for Zn (II) ions of 272.27 mg/g at a pH of Zn (II) initial solution of 6. The sorption capacity was 1.5 higher in slightly acidic conditions (pH 6) comparative with that for strong acidic conditions (pH 3). The sorption isotherms were consistent with the Langmuir model and the sorption kinetics was well fitted by a pseudo-second order kinetic model. The intraparticle diffusion was considered to be the rate-determining step for Zn (II) sorption. Two main sorption mechanisms for Zn (II) were identified, namely ion exchange and chemisorption (surface complexation). Therefore, it could be concluded that the new GG/GO composite is a feasible sorbent for the retention of Zn (II) from aqueous solutions under certain pH conditions. Given the promising sorption capacity of the new composite hydrogel obtained for zinc ions, it is envisaged to carry out further experiments that will target the mechanical stability of this material in both static and dynamic sorption conditions, as well as the extension of experiments for other heavy metal ions.

## Figures and Tables

**Table 1:** Elements percentages derived from XPS analysis.

Sample	C 1s (%)	O 1s (%)	Zn 2p (%)
GG GG/Zn^2+^ GG/GO GG-GO/Zn^2+^	69.88 75.22 62.02 78.32	30.12 21.04 31.31 20.33	0 1.24 0 1.35

**Table 2:** Fitting parameters for Langmuir and Freundlich isotherms of Zn (II) sorbed on GG and GG/GO.

Sorbent	Parameters					
	Langmuir			Freundlich		
	q_m_ (mg/g)	K_L_ (L/mg)	R^2^	1/n	K_F_ (mg^1−n.^ L^n^/g)	R^2^
GG; pH 6 GG/GO; pH 6 GG; pH 3 GG/GO; pH 3	120.48 272.27 84.03 178.57	0.043 0.116 0.028 0.096	0.9946 0.9935 0.9921 0.9914	0.573 0.575 0.583 0.545	9.057 32.564 5.015 20.306	0.9798 0.9841 0.9471 0.9675

**Table 3:** Sorption capacity of different materials for Zn (II). Values were obtained in the best operation conditions.

Sorbent	Sorption Capacity, mg/g	Reference
Activated Carbon (adsorbent dose 0.5 g/L, pH 6.5)	40.86	[38]
Nanoporous carbon (adsorbent dose 0.5 g/L, pH 6.5)	130.76	
Graphene oxide (adsorbent dose 2 mg/mL, pH 7)	246.00	[39]
Graphene oxide (adsorbent amount 2.5 mg, GO 0.0025 ppm, pH 7)	287.86	[40]
Graphene oxide-Polyaniline (adsorbent amount 2.5 mg, GO 2.5 mg, pH 7)	297.62	
Silk fibroin/polyethylenimine hydrogel (adsorbent dose 0.5 g/L, pH 5.5)	125.00	[41]
Chitosan, itaconic acid, and methacrylic acid based hydrogel (adsorbent amount 0.035 g, pH 5.5)	105.50	[42]
Poly(acrylate-acrylic acid-co-maleic acid) and poly(2-acrylamido-2-methyl-1-propanesulfonic acid) based hydrogel (adsorbent amount 0.01 g)	267.73	[43]
Magnetic Poly(acrylate-acrylic acid-co-maleic acid) and poly(2-acrylamido-2-methyl-1-propanesulfonic acid) based hydrogel (adsorbent amount 0.01 g)	289.12	
Gellan Gum (liquid-to-solid mass ratio of 50/1, pH 6)Gellan Gum/Graphene oxide 1 wt % (liquid-to-solid mass ratio of 50/1, pH 6)	120.48272.27	This article

**Table 4:** Kinetic parameters and the correlation coefficients of Zn (II) sorption on GG and GG/GO.

Kinetic Model	Parameters	GG pH 6	GG/GO pH 6	GG pH 3	GG/GO pH 3
Pseudo-first order	q_e_ k_1_, min^−1^ R^2^	163.47 0.155 0.9671	207.05 0.132 0.8832	38.16 0.147 0.8580	76.52 0.143 0.9623
Pseudo-second order	q_e_ k_2_, g mg^−1^ min^−1^ R^2^	156.25 0.0045 0.9919	250.00 0.0040 0.9917	49.02 0.0012 0.9923	103.09 0.0017 0.9958
Intraparticle diffusion (Stage 1)	k_id1,_ mg g^−1^ min^−1/2^ C_1_ R^2^	31.544 8.167 0.9596	43.488 44.513 0.9555	7.637 8.993 0.9759	14.124 29.962 0.9623
Intraparticle diffusion (Stage 2)	k_id2,_ mg g^−1^ min^−1/2^ C_2_ R^2^	8.911 83.143 0.9297	6.440 192.89 0.8932	0.766 40.766 0.6950	10.532 87.400 0.9948
